# Supplementary material for: Whole-Genome Sequencing of a Colistin-Resistant Acinetobacter baumannii Strain Isolated at a Tertiary Health Facility in Pretoria, South Africa
Source: Antibiotics (Basel). 2022 Apr 28;11(5):594. doi: 10.3390/antibiotics11050594 (PMC9138137; doi:10.3390/antibiotics11050594)
Supplement: Supplementary file 1 [file antibiotics-11-00594-s001.zip › antibiotics-1658602-supplementary.pdf]

Supplemantary Materials

Table S1: Primers sequences used for PCR

PRIMERS	SEQUENCES	GENES	SIZES [REFERENCES]
OXA-51 for<br>OXA-51 rev	TAATGCTTTGATCGGCCTTG<br>TGGATTGCACTTCATCTTGG	blaOXA-51	353bp [1]
AdeB for<br>AdeB rev	TTAACGATAGCGTTGTAACC<br>TGAGCAGACAATGGAATAGT	AdeB	541bp [2]
AdeR for<br>AdeR rev	ACTACGATATTGGCGACATT<br>GCGTCAGATTAAGCAAGATT	AdeR	447bp [2]
AdeS for<br>AdeS rev	TTGGTTAGCCACTGTTATCT<br>AGTGGACGTTAGGTCAAGTT	AdeS	544bp [2]
MCR for<br>MCR rev	CGGTCAGTCCGTTTGTTC<br>CTTGGTCGGTCTGTAGGG	mcr-1	309bp [3]

Table S2: Thermocycling condition used in the study

TARGETED GENES	THERMOCYCLING CONDITION	POSITIVE CONTROL
bla$_{OXA-51}$	94 °C for 4 min<br>35 X $\begin{cases} 94\ °C \text{ for } 30 \text{ sec} \\ 50\ °C \text{ for } 30 \text{ sec} \\ 72\ °C \text{ for } 1 \text{ min} \end{cases}$<br>72 °C for 90 sec	*A. baumannii* ATCC® BAA-1605™ strain<br>*K. pneumoniae* ATCC 8303 [Klebsiella pneumoniae carbapenemase (KPC) positive control]<br>*K. pneumoniae* ATCC BAA-2146 [New Delhi metallo-β-lactamase (NDM) positive control]
adeS<br>*adeR*	94 °C for 5 min<br>30 X $\begin{cases} 94\ °C \text{ for } 1 \text{ min} \\ 57\ °C \text{ for } 1 \text{ min} \\ 72\ °C \text{ for } 1 \text{ min} \end{cases}$<br>72 °C for 5 min	
adeB	94 °C for 5 min<br>30 X $\begin{cases} 94\ °C \text{ for } 1 \text{ min} \\ 57\ °C \text{ for } 1 \text{ min} \\ 72\ °C \text{ for } 1 \text{ min} \end{cases}$<br>72 °C for 5 min	
	RT-PCR	
mcr-1	95 °C for 5 min<br>35 X $\begin{cases} 95\ °C \text{ for } 30\text{s} \\ 61.2°C \text{ for } 40\text{s} \\ 72\ °C \text{ for } 40\text{s} \end{cases}$<br>70 °C for 10 min	N/A

*min: Minute (s)

*sec: Second (s)

*N/A: Not applicable

REFERENCES

1 Kock MM, Bellomo AN, Storm N, Ehlers MM. Prevalence of carbapenem resistance genes in Acinetobacter baumannii isolated from clinical specimens obtained from an academic hospital in South Africa. Southern African Journal of Epidemiology and Infection. 2013 Jan 1;28(1):28-32. https://doi.org/10.1080/10158782.2013.11441516

2 Beheshti M, Talebi M, Ardebili A, Bahador A, Lari AR. Detection of AdeABC efflux pump genes in tetracycline-resistant Acinetobacter baumannii isolates from burn and ventilator-associated pneumonia patients. Journal of pharmacy & bioallied sciences. 2014 Oct;6(4):229. doi: 10.4103/0975-7406.142949

3 Hazrat B, Fareeha H, Muhammad KA, Sabir K, Xingyuan Y, Tayyab RU. Detection of mcr-1 gene in extended-spectrum β-lactamase-producing Klebsiella pneumoniae from human urine samples in Pakistan. Jundishapur Journal of Microbiology. 2020; 13(4), pp. 1–6. doi: 10.5812/jjm.96646.
